# MtnBD Is a Multifunctional Fusion Enzyme in the Methionine Salvage Pathway of *Tetrahymena thermophila*

**DOI:** 10.1371/journal.pone.0067385

**Published:** 2013-07-01

**Authors:** Toshihiro Nakano, Izuru Ohki, Akiho Yokota, Hiroki Ashida

**Affiliations:** 1 Graduate School of Biological Sciences, Nara Institute of Science and Technology (NAIST), Ikoma, Nara, Japan; 2 Japan Science and Technology Agency (JST), Precursory Research for Embryonic Science and Technology (PRESTO), Kawaguchi, Saitama, Japan; University of Florida, United States of America

## Abstract

To recycle reduced sulfur to methionine in the methionine salvage pathway (MSP), 5-methylthioribulose-1-phosphate is converted to 2-keto-4-methylthiobutyrate, the methionine precursor, by four steps; dehydratase, enolase, phosphatase, and dioxygenase reactions (catalyzed by MtnB, MtnW, MtnX and MtnD, respectively, in *Bacillus subtilis*). It has been proposed that the MtnBD fusion enzyme in *Tetrahymena thermophila* catalyzes four sequential reactions from the dehydratase to dioxygenase steps, based on the results of molecular biological analyses of mutant yeast strains with knocked-out MSP genes, suggesting that new catalytic function can be acquired by fusion of enzymes. This result raises the question of how the MtnBD fusion enzyme can catalyze four very different reactions, especially since there are no homologous domains for enolase and phosphatase (MtnW and MtnX, respectively, in *B. subtilis*) in the peptide. Here, we tried to identify the domains responsible for catalyzing the four reactions using recombinant proteins of full-length MtnBD and each domain alone. UV-visible and ^1^H-NMR spectral analyses of reaction products revealed that the MtnB domain catalyzes dehydration and enolization and the MtnD domain catalyzes dioxygenation. Contrary to a previous report, conversion of 5-methylthioribulose-1-phosphate to 2-keto-4-methylthiobutyrate was dependent on addition of an exogenous phosphatase from *B. subtilis*. This was observed for both the MtnB domain and full-length MtnBD, suggesting that MtnBD does not catalyze the phosphatase reaction. Our results suggest that the MtnB domain of *T. thermophila* MtnBD acquired the new function to catalyze both the dehydratase and enolase reactions through evolutionary gene mutations, rather than fusion of MSP genes.

## Introduction

Duplication, fusion, mutation, and homologous recombination of genes encoding enzymes have contributed to the emergence, diversification, and development of metabolic pathways during evolution [1]–[3]. Recent progress in genome analysis has shown that genes encoding proteins of known function may be found in other species as fusion genes encoding a single multifunctional protein [4]–[7]. This event, known as gene fusion/protein fusion, is one of several molecular evolutionary processes [8], [9]. In prokaryotes, biosynthetic reactions are generally catalyzed by a series of mono-functional enzymes. In contrast, higher eukaryotes often utilize multifunctional fusion enzymes that catalyze several biosynthetic steps. This trend has been observed for metabolic enzymes catalyzing fatty acid and purine biosynthesis [10], [11]. The advantages of fusion enzymes include decreased regulatory load on metabolic pathways and enhanced catalytic efficiency of sequential reactions without the loss of metabolic intermediates.

**Figure 1:**
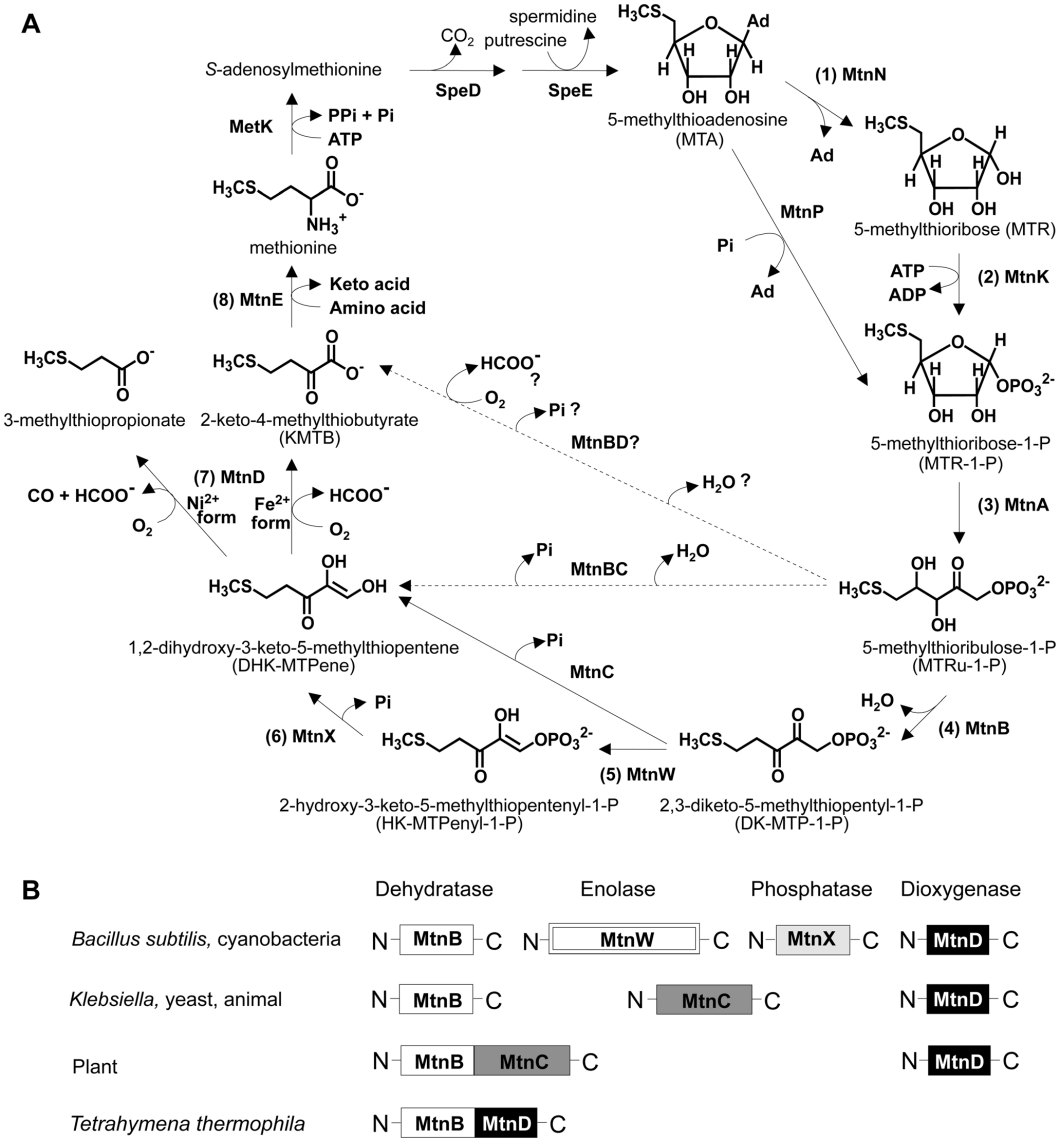
Methionine salvage pathway (MSP). (A) MSP in *B. subtilis* and other organisms. Enzyme names are derived from Ashida et al. [19] and Salim et al. [14]. The MSP recycles organic sulfur from MTA, a by-product of polyamine synthesis, to methionine. MTA phosphorylase (MtnP) in mammals and yeasts catalyzes a one-step reaction to yield MTR-1-P from MTA. In almost all microorganisms, plants, and specific protozoa except for *T. thermophila*, this phosphorylase is replaced by two enzymes; MTA nucleosidase (MtnN) and MTR kinase (MtnK). (B) Diversity of MSP enzymes among various organisms. In *B. subtilis*, the MSP consists of eight enzymes because it has one enzyme for each reaction. While *B. subtilis* utilizes two separate enzymes (MtnW and MtnX) to catalyze the enolization and dephosphorylation steps, in most living organisms including proteobacteria, yeasts, plants, and animals, DK-MTP-1-P is converted into HK-MTPenyl-1-P by a bi-functional DK-MTP-1-P enolase/phosphatase (MtnC) belonging to the haloacid dehalogenase superfamily. In plants and *T. thermophila,* MtnB is fused with MtnC and MtnD, respectively.

It has been predicted that *Tetrahymena thermophila* has 52 genes for fusion enzymes [12] because the UAA and UAG stop codons are translated as glutamine in this organism [13]. Gene fusions of *mtnA* and *mtnK*, and *mtnB* and *mtnD*, which encode enzymes in the methionine salvage pathway (MSP), are present in the genome of *T. thermophila* [14]. MSP is a universal pathway in all kingdoms [15]; it recycles reduced sulfur from 5-methylthioadenosine (MTA), a by-product of polyamine synthesis, to methionine (Fig. 1A) [15]–[17]. The bacterial MSP has been well studied in *Bacillus subtilis* (Fig. 1A) [18]–[23]. In this one-enzyme–one-reaction pathway of *B. subtilis*, MTA is first hydrolyzed into 5-methylthioribose (MTR) and adenine by MTA nucleosidase (MtnN) (step 1) [23], [24]. Next, MTR is phosphorylated to form MTR-1-phosphate (MTR-1-P) by the MTR kinase (step 2) [25]–[27], and then MTR-1-P is isomerized by MTR-1-P isomerase (MtnA) to form 5-methylthioribulose-1-phosphate (MTRu-1-P) (step 3) [20], [28], [29]. MTRu-1-P undergoes a dehydration step catalyzed by MTRu-1-P dehydratase (MtnB), producing 2,3-diketo-5-methylthiopentyl-1-phosphate (DK-MTP-1-P) (step 4) [18], [28], [30]. Then, DK-MTP-1-P is enolized to form 2-hydroxy-3-keto-5-methylthiopentenyl-1-phosphate (HK-MTPenyl-1-P) by DK-MTP-1-P enolase (MtnW) (step 5) [19], [21], [31], [32]. Subsequently, HK-MTPenyl-1-P is dephosphorylated and converted into 1,2-dihydroxy-3-keto-5-methylthiopentene (DHK-MTPene) by HK-MTPenyl-1-P phosphatase (MtnX) [18], a *Bacillus*-specific enzyme (step 6). DHK-MTPene is converted into 2-keto-4-methylthiobutyrate (KMTB) and formate by a DHK-MTPene dioxygenase (MtnD) (step 7) [18], [23], and KMTB is finally transaminated to form methionine by KMTB aminotransferase (MtnE) (step 8) [33].

When reactions from MTRu-1-P to KMTB (steps 4 to 7) are compared among various organisms, they are catalyzed by four enzymes in Bacilli, three enzymes in pathogenic *Klebsiella oxytoca*, yeast, and mammals, two enzymes in plants, and probably one enzyme in *T. thermophila* (Fig. 1B) [14], [18]–[21], [30], [34]–[36]. Thus, the MSP is catalyzed by different enzyme combinations in different organisms, despite going through the same metabolites. Multifunctional MtnC and MtnBD contribute to diversification of the MSP. MtnC is a bi-functional enzyme that acts as a DK-MTP-1-P enolase/phosphatase, catalyzing steps 5 and 6 in the MSP (Fig. 1A) [37]–[39]. In terms of multifunctionality, the most notable enzyme is *T. thermophila* MtnBD, the fusion protein of MtnB and MtnD. This enzyme probably catalyzes the four reaction steps of MtnB, MtnW, MtnX, and MtnD, based on the results of complementation experiments with yeast knockout mutants of MSP genes [14]. Salim and co-workers proposed that the fusion of proteins is an evolutionary mechanism to gain a new function and has reduced the number of enzymes in the MSP of *T. thermophila*.

Here, we used biochemical approaches to analyze the catalytic properties of the *T. thermophila* MtnBD fusion enzyme. We investigated four catalytic reactions of *T. thermophila* MtnBD using recombinant proteins for full-length MtnBD and the MtnB domain only. The results showed that the MtnD domain of the *T. thermophila* MtnBD catalyzes the DHK-MTPene dioxygenase reaction, and the MtnB domain catalyzes the DK-MTP-1-P enolase reaction in addition to the MTRu-1-P dehydratase reaction. This suggests that the MtnBD of *T. thermophila* obtained new enolization catalytic function through the molecular evolution of the MtnB domain, rather than through protein fusion.

## Materials and Methods

### Design of the *Tetrahymena mtnBD* Gene

*T. thermophila* translates UAA and UAG as glutamine instead of stop codons [13]. These codons on the *Tetrahymena mtnBD* gene (Genbank accession number XM_001025046) were replaced by CAA or CAG codons for glutamine and other codons were modified to expression in *Escherichia coli* (Fig. S1). *Nde*I (CATATG) and *Bam*HI (GGATCC) sites were attached to the 5′ and 3′ ends of the gene. The designated gene was purchased from GenScript Corporation (Piscataway, NJ). The synthesized gene (syn_ttmtnBD) was inserted into the *Nde*I-*Bam*HI site of the pET-15b vector (Novagen, Madison, WI), producing pET15b-ttmtnBD.

### Design of *Tetrahymena mtnB* (encoding Met1–Ser216) and *mtnD* (encoding Ser217–Lys400) Genes

The sequence for the MtnD domain was deleted by polymerase chain reaction (PCR) with KOD DNA Polymerase plus (Toyobo, Tokyo, Japan) using pET15b-ttmtnBD as the template. The resulting vector was named pET15b-ttmtnB and expressed only MtnB. The thermal cycle program was as follows: denaturation at 94°C for 2 min, followed by 30 cycles of denaturation at 94°C for 30 s, annealing at 55°C for 50 s, and elongation at 68°C for 395 s. The PCR primers were ttmtnD-del-f (5′-TAAGGATCCGGCTGCTAACAAAG-3′) and ttmtnD-del-r (5′-AGAGCTCACGGTGCGCGGAATTT-3′). 5′-*Nde*I and 3′-*Bam*HI sites were attached to the sequence of the *mtnD* domain (coding Ser217–Lys400), and *mtnD* was amplified by PCR with pET15b-ttmtnBD as the template. The resulting *mtnD* gene sequence was inserted into the *Nde*I-*Bam*HI region of pET-15b, yielding pET15b-ttmtnD. The PCR conditions for amplifying *mtnD* were similar to those for pET15b-ttmtnB, except that the elongation step was 35 s at 68°C. The primers were ttmtnD-*Nde*I-f (5′-GCATATGAGTTCCCAGCTGCGTGCATGG-3′) and ttmtnD-*Bam*HI-r (5′-GTTAGCAGCCGGATCCTTATTTTTGG-3′).

### Expression, Purification, and Polyacrylamide Gel Electrophoresis (PAGE) Analysis of Full-length MtnBD and MtnB Domain Proteins

Histidine-tagged recombinant proteins of full-length MtnBD and the MtnB domain were expressed in *E. coli* BL21 (DE3) (Novagen) at 25°C for 12 h in LB medium containing 50 µg mL^−1^ ampicillin and 1 mM isopropyl β-d-thiogalactopyranoside. *E. coli* cells were harvested by centrifugation at 5,500×*g*. MtnBD and MtnB domain proteins were purified from cell extracts using His-Bind resin (Novagen) as described previously [18], [40]. MtnK for synthesis of MTR-1-P, and MtnA and MtnX from *B. subtilis* were expressed in *E. coli* BL21 (DE3) and purified by Ni affinity chromatography [18], [20]. Native and sodium dodecyl sulfate (SDS) PAGE were carried out as described previously [41]. We purchased 5–20% gradient polyacrylamide gels from ATTO (Tokyo, Japan), the high molecular weight calibration kit for native-PAGE from GE Healthcare (Waukesha, WI), and denatured pre-stained protein markers (Venus markers) from Nacalai (Kyoto, Japan).

### Synthesis and Purification of MTR-1-P

MTR-1-P was synthesized using purified *B. subtilis* MtnK and MTR obtained by acid hydrolysis of *S*-adenosylmethionine [20]. A mixture of 1 mg MtnK in 50 mL 100 mM Tris-HCl (pH 8.0), 5 mM MgCl_2_, 5 mM MTR, and 8 mM ATP was incubated overnight at 37°C for enzymatic synthesis of MTR-1-P. The reaction mixture containing MTR-1-P was loaded onto a column (2.5 cm i. d. ×30 cm) containing 50 mL activated charcoal (Wako, Osaka, Japan) to remove ATP and ADP. MTR-1-P was eluted with 1 L 5% ethanol. Fractions containing MTR-1-P were identified by the reducing sugars assay [42]. The solution was evaporated and the residue was dissolved in H_2_O and then loaded onto a HR 5/50 MonoQ column (GE Healthcare) for further purification. MTR-1-P was eluted from the column with a linear gradient (20 mL) from 0 to 200 mM NaCl at 1 mL min^−1^. MTR-1-P eluted at 10 to 50 mM NaCl. The product-containing fractions were detected by the reducing sugars assay and the inorganic phosphate assay (Phosphor C, Wako).

### Assay of MTRu-1-P Dehydratase/Enolase Activity

The MTRu-1-P dehydratase/enolase activities of MtnBD and the MtnB domain were estimated using the molecular extinction coefficient of HK-MTPenyl-1-P (9,500 M^−1^ cm^−1^ at 280 nm) [20], [21], [30]. The reaction was initiated by adding 2.2 µg MtnBD or 2.4 µg MtnB domain proteins to 100 µL 50 mM Tris-HCl (pH 8.0), 5 mM MgCl_2_, 100 µM MTR-1-P, and 9 µg MtnA at 35°C. MTR-1-P was isomerized to MTRu-1-P beforehand by MtnA at 35°C.

### ^1^H-nuclear Magnetic Resonance (NMR) Analysis

^1^H-NMR spectra were recorded at 298 K using a Bruker DRX-800 spectrometer (Bruker BioSpin, Yokohama, Japan) operated at 800 MHz. Lyophilized MTR-1-P as the starting substrate was dissolved in 99.9% D_2_O (Euriso-top, Gif-sur-Yvette, France) containing 25 mM sodium phosphate (pD 7.5) and 0.1 mM MgCl_2_. To remove H_2_O from the enzyme purification buffer, the enzyme solution was passed through a NAP-5 column (GE Healthcare) equilibrated with the same buffer. MTR-1-P:^ 1^H-NMR (800 MHz) δ 5.53 (dd, *J* = 4.4, 6.0 Hz, H1), 4.24 (dd, J = 5.4, 10.6 Hz, H2), 4.07 (m, H3), 3.93 (m, H4), 2.75–2.63 (ABX, *J* = 6.0, 13.9, 71.8 Hz, H5), 2.09 (s, H6). MTRu-1-P: δ 4.65 (ABX, J = 6.2, 18.8, 44.4Hz, H1), 4.46 (d, J = 5.0 Hz, H3), 4.11–4.09 (m, H4), 2.71 (dd, J = 4.5, 14.1 Hz, H5A), 2.62 (dd, J = 8.2, 14.0 Hz, H5B), 2.07 (m, H6). HK-MTPenyl-1-P: δ 7.48 (d, J = 8.2 Hz, H1), 2.89 (t, J = 6.7 Hz, H4), 2.76 (d, J = 7.0 Hz, H5), 2.07 (s, H6). DHK-MTPene: δ 8.50 (s, H1), 2.73 (d, J = 7.1 Hz, H5), 2.67 (t, J = 6.7 Hz, H4), 2.06 (s, H6). A deuterium was incorporated at the C4 positions of HK-MTPenyl-1-P and DHK-MTPene.

### High Performance Liquid Chromatography (HPLC) Analysis

The MtnBD reaction products were analyzed using a Class VP HPLC system (Shimadzu, Kyoto, Japan) [18]. A mixture of 300 µL 50 mM Tris-HCl (pH 8.0), 5 mM MgCl_2_, 1 mM MTR-1-P, 50 µg *B. subtlis* MtnA, 60 µg MtnBD, and 6 µg *B. subtilis* MtnX was incubated for 15 h at 25°C. All enzymes were removed by filtration through a Microcon Ultracel YM-10 (NMWL 10,000, Millipore, Billerica, MA) at 14,000×*g* for 40 min. An aliquot (25 µL) of filtered solution was injected into the HPLC. The 25 µL samples of reaction product or 1 mM authentic KMTB (Sigma) were separated on an Aminex HPX-87 column (Bio-Rad, Richmond, CA) with 5 mM H_2_SO_4_ and 20% acetonitrile at 0.6 mL min^−1^ and 60°C. KMTB was monitored by measuring the absorbance at 210 nm.

## Results

### Full-length *Tetrahymena* MtnBD Catalyzes MTRu-1-P Dehydratase/enolase and DHK-MTPene Dioxygenase Reactions

While MtnBD shows no similarity to MtnW (DK-MTP-1-P enolase), MtnX (HK-MTPenyl-1-P phosphatase), or MtnC (DK-MTP-1-P enolase/phosphatase), previous genetic experiments suggested that it catalyzes four sequential reactions; dehydration, enolization, dephosphorylation, and dioxygenation [14]. To analyze whether MtnBD catalyzes these four reactions (steps 4 to 7) *in vitro*, we designed a recombinant protein of *T. thermophila* MtnBD fused with a histidine-tag at the N-terminus (Fig. S2A). MtnBD was expressed as a soluble protein by *E. coli* BL21 (DE3), and the purified enzyme was confirmed as a single band on an SDS-PAGE gel (Fig. S2B). Native-PAGE analysis showed that the molecular mass of MtnBD (calculated molecular mass of a monomer = 48.4 kDa) was approximately 290 kDa (Fig. S2D), suggesting that MtnBD formed a homohexamer.

**Figure 2:**
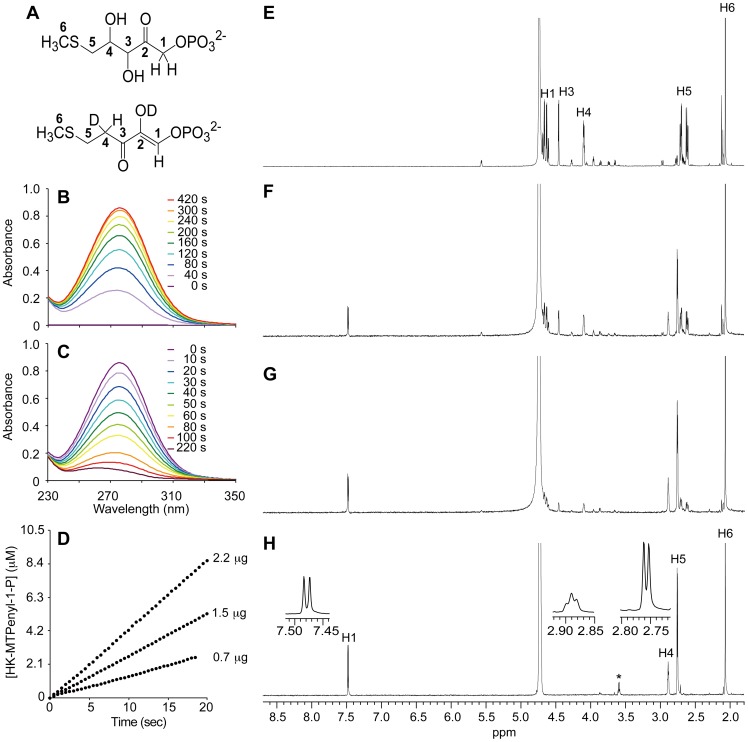
UV-visible and ^1^H-NMR spectra of metabolites synthesized by *Tetrahymena* MtnBD. (A) Conversion of MTRu-1-P into HK-MTPenyl-1-P in D_2_O phosphate buffer. Dehydration of MTRu-1-P (upper part) may introduce a proton from solvent into C4 of HK-MTPenyl-1-P (lower part). (B) UV-visible spectral changes after adding MtnBD into reaction mixture containing MTRu-1-P. Spectra are shown in different colors (0, 40, 80, 120, 160, 200, 240, 300, and 420 s). (C) Spectral changes before and after adding 0.14 µg *B. subtilis* MtnX to reaction product in Fig. 2B. Spectra are shown in different colors (0, 10, 20, 30, 40, 50, 60, 80, 100, and 220 s). In (B) and (C), spectra of products were measured at 35°C in 100 µL 50 mM Tris-HCl (pH 8.0), 5 mM MgCl_2_, 100 µM MTR-1-P, and 9 µg *Bacillus* MtnA. MTR-1-P was converted into MTRu-1-P by MtnA before assay. (D) Time course of HK-MTPenyl-1-P production with different amounts of MtnBD protein. The reaction was initiated by adding 0.7, 1.5 or 2.2 µg MtnBD proteins to 100 µL 50 mM Tris-HCl (pH 8.0), 5 mM MgCl_2_, 100 µM MTR-1-P, and 9 µg MtnA at 35°C. MTR-1-P was converted into MTRu-1-P by MtnA before assay. Concentration of HK-MTPenyl-1-P was estimated using the molecular extinction coefficient (9,500 M^−1^ cm^−1^ at 280 nm). (E) ^1^H-NMR spectrum of product (MTRu-1-P) from *B. subtilis* MtnA. Isomerization reaction in 250 µL 25 mM sodium phosphate (pD 7.5), 0.1 mM MgCl_2_, 1 mM MTR-1-P, and 20 µg *B. subtilis* MtnA at 37°C for 30 min. (F to H) ^1^H-NMR spectra after adding 10 µg MtnBD into reaction mixture containing MTRu-1-P. * indicates chemical shift associated with degraded compound (elimination of methyl group at C1). Reaction products generated in magnesium phosphate buffer at 25°C and pD 7.5 at 1 h (F), 2 h (G) and 6 h (H). ^1^H peak at 4.74 ppm represents proton from residual H_2_O.

The reactions catalyzed by the full-length *T. thermophila* MtnBD were determined by analyzing UV-visible and ^1^H-NMR spectra of the reaction products (Fig. 2). The MSP metabolites DK-MTP-1-P, HK-MTPenyl-1-P, and DHK-MTPene show specific absorbance at 270, 278, and 310 nm, respectively [18], [21], [30]. To identify the product, we analyzed the UV-visible spectral changes during the reaction of *T. thermophila* MtnBD with MTRu-1-P (Fig. 2B). Addition of MtnBD to a reaction mixture containing MTRu-1-P resulted in an increase in absorbance at 278 nm (Fig. 2B). The doublet peak at 3.9 ppm for the predicted C1-proton of DK-MTP-1-P, a dehydratase reaction product, was not observed in the ^1^H-NMR spectra during the MtnBD reaction (Fig. 2E–H) [28]. However, the C1-proton of HK-MTPenyl-1-P as a doublet peak at 7.48 ppm appeared after 1 h of the MtnBD reaction (Fig. 2E, F). After 2 h of the reaction, a doublet peak at 2.76 and a broad triplet peak at 2.89 ppm appeared and were assigned as C5- and C4-protons of HK-MTPenyl-1-P, respectively (Fig. 2G), the same as the spectrum previously reported for HK-MTPenyl-1-P [18]. ^1^H-NMR analysis clearly showed that the *T. thermophila* MtnBD catalyzed the conversion of MTRu-1-P to HK-MTPenyl-1-P. The production rate of HK-MTPenyl-1-P depended on the amount of MtnBD protein (Fig. 2D). The ^1^H-NMR spectra of HK-MTPenyl-1-P showed that a deuterium was incorporated at the C4 position of HK-MTPenyl-1-P during the reaction catalyzed by *T. thermophila* MtnBD (Fig. 2A, F, G, H). In our previous study, a C4-proton of HK-MTPenyl-1-P was replaced with a deuterium during the dehydration of MTRu-1-P by *B. subtilis* MtnB before DK-MTP-1-P enolization [18]. These results suggested that the reaction of *T. thermophila* MtnBD proceeds through the MTRu-1-P dehydration in a similar manner to that of the *B. subtilis* MtnB. ^1^H-NMR analysis showed that MTRu-1-P was completely converted into HK-MTPenyl-1-P after 6 h (Fig. 2H). The spectrum did not change after 15 h (data not shown). These results suggested that *T. thermophila* MtnBD catalyzes the MTRu-1-P dehydration/enolization, but not the dephosphorylation of HK-MTPenyl-1-P.

Addition of *B. subtilis* MtnX (HK-MTPenyl-1-P phosphatase) into the reaction mixture containing HK-MTPenyl-1-P produced by *T. thermophila* MtnBD resulted in a spectral change with a decrease in absorption at 278 nm of HK-MTPenyl-1-P. Whereas the *B. subtilis* MtnX converted HK-MTPenyl-1-P to DHK-MTPene, as we reported previously [18], there was neither a spectral shift with an isosbestic point at 289 nm nor an increase in specific absorption at 310 nm indicating accumulation of DHK-MTPene (Fig. 2C). The lack of DHK-MTPene production suggested that there was a subsequent rapid dioxygenation of DHK-MTPene catalyzed by the MtnD domain of *T. thermophila* MtnBD after the dephosphorylation reaction of *B. subtilis* MtnX. This result indicated that the *T. thermophila* MtnBD also catalyzes the dioxygenation of DHK-MTPene.

### MtnB Domain for MTRu-1-P Dehydration and DK-MTP-1-P Enolization

**Figure 3:**
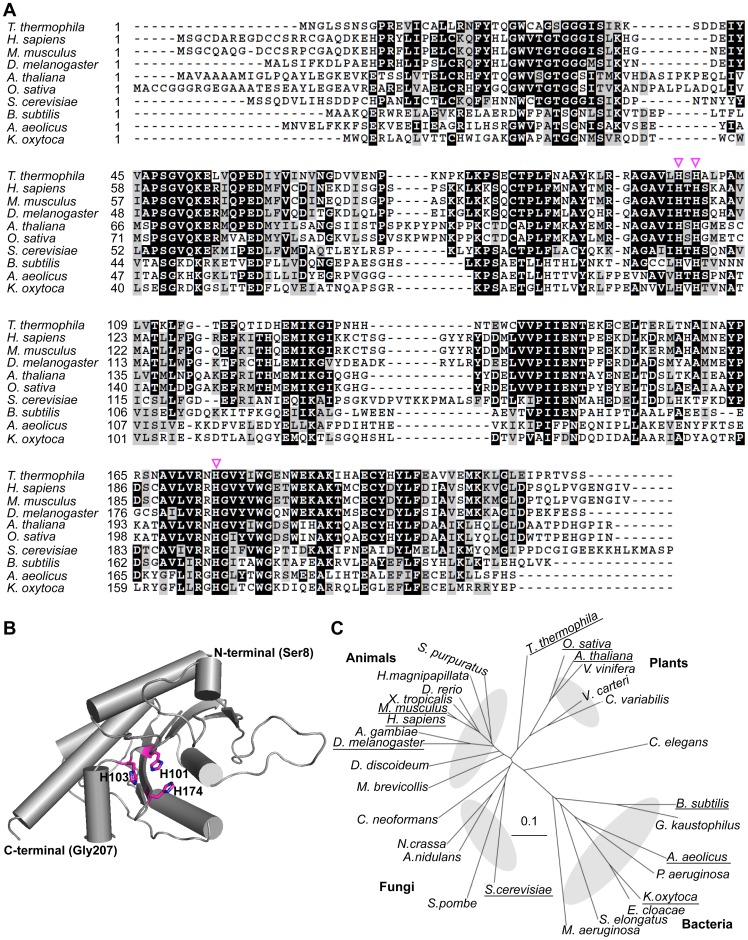
Sequence, structure, and phylogenetic analyses of various MtnBs. (A) Multiple sequence alignment of MtnBs. Magenta triangles indicate histidine residues essential for binding divalent metal ion. Numbers on left of sequences show innate amino acid number for each protein. The alignment was created using ClustalW; identical and similar amino acids were highlighted/shaded with Boxshade. (B) Predicted tertiary structure of the *T. thermophila* MtnB domain. This was predicted from the known structure of *A. aeolicus* MtnB (PDB ID: 2IRP) using Swiss-model (http://swissmodel.expasy.org/). Three histidine residues essential for metal binding are shown by magenta sticks. Nitrogen atoms of histidine residues at active site are shown in blue. Structure was drawn using PyMOL version 0.98 (http://pymol.org). (C) Phylogenetic tree based on primary sequences of MtnB family. Alignments were created with ClustalW and displayed using Treeview. Scale bar indicates difference of 0.1 substitutions per site. Full names and gene accession numbers are as follows: *Anopheles gambiae* str. PEST (XP_310624), *A. aeolicus* VF5 (NP_214357), *Arabidopsis thaliana* (NP_974931 residues 1–247), *Aspergillus nidulans* FGSC A4 (XP_661197), *B. subtilis* str 168 (NP_389244), *Caenorhabditis elegans* (NP_509690), *Chlorella variabilis* (EFN54454 residues 1–254), *Cryptococcus neoformans* var. *neoformans* JEC21 (XP_572402), *Danio rerio* (NP_001004679), *Dictyostelium discoideum* (XP_639930), *Drosophila melanogaster* (NP_572916), *Enterobacter cloacae* subsp. *cloacae* ATCC 13047 (YP_003613571), *Geobacillus kaustophilus* HTA426 (YP_146808), *Homo sapiens* (NP_057041), *Hydra magnipapillata* (XP_002165198), *Klebsiella oxytoca* (formerly *Klebsiella pneumoniae* 342) (YP_002239745), *Microcystis aeruginosa* PCC7806 (CAO89699), *Monosiga brevicollis* MX1 (XP_001750472), *Mus musculus* (NP_062709), *Neurospora crassa* OR74A (XP_964699), *Oryza sativa* Japonica Group (NP_001067908 residues 1–252), *Pseudomonas aeruginosa* PAO1 (NP_250374), *Saccharomyces cerevisiae* S288c (NP_012558), *Schizosaccharomyces pombe* 972h- (NP_593625), *Strongylocentrotus purpuratus* (XP_794552 residues 3114–3362), *Synechococcus elongatus* PCC 6301 (YP_172813), *Vitis vinifera* (XP_002274553 residues 1–257), *Volvox carteri* f. *nagariensis* (XP_002956646 residues 1–218), *Xenopus tropicalis* (NP_001015712). Although some MtnBs consist of more than two domains, only MtnB domains were used for phylogenetic analysis. Proteins described in (A) are underscored.

In the primary sequence of *T. thermophila* MtnBD, the N-terminal amino acid sequence (1–216) showed 50.2, 39.2, and 26.7% identity with MtnBs of *Homo sapiens*, *Saccharomyces cerevisiae,* and *B. subtilis,* respectively. The C-terminal amino acid sequence (217–400) of *T. thermophila* MtnBD showed 49.5, 53.3, 38.6, 47.9, and 25.0% identity with MtnDs of *H. sapiens*, *Mus musculus*, *S. cerevisiae*, *Arabidopsis thaliana*, and *B. subtilis*, respectively. However, there was neither a MtnW domain with a catalytic KDDE motif, nor a MtnC domain with three catalytic motifs [37], in the *T. thermophila* MtnBD. To determine whether protein fusion was essential for the enolase reaction or which domain in the *T. thermophila* MtnBD was responsible, we tried to analyze the enzymatic activities of two recombinant proteins, one consisting of the MtnB domain and the other the MtnD domain. To identify the MtnB domain of *T. thermophila* MtnBD for expression in *E. coli*, we conducted structural homology modeling and sequence analysis of the MtnB domain by comparing it with known prokaryotic and eukaryotic MtnBs (Fig. 3A). The MtnB domain of *T. thermophila* MtnBD had three conserved histidine residues: His101, His103, and His174, which coordinate an essential metal ion in other MtnBs. Homology modeling of the *T. thermophila* MtnB domain using *Aquifex aeolicus* MtnB (PDB ID: 2IRP) as the template suggested that these three active-site histidine residues probably form a catalytic center in the predicted structure of the MtnB domain (Fig. 3B). In the phylogenetic tree of MtnBs and the *T. thermophila* MtnB domain, MtnBs were classified into four clades; animals, plants, fungi, and bacteria. The MtnB domain of *T. thermophila* MtnBD was grouped with MtnB domains of plant-type MtnBC fusion enzymes (Fig. 3C). When the amino acid sequence of the *T. thermophila* MtnBD was compared with the C-terminal regions of MtnBs from other organisms, the MtnB domain of *T. thermophila* MtnBD showed higher identity with the C-terminal sequence of *Drosophila melanogaster* MtnB than to other eukaryotic MtnBs. From a sequence alignment with *D. melanogaster* MtnB, we determined that Met1–Ser216 represented the MtnB domain and Ser217–Lys400 the MtnD domain; therefore, these sequences were used to design recombinant proteins for each domain from *T. thermophila* MtnBD (Fig. S2A). The *T. thermophila* MtnB domain fused with a histidine-tag was expressed in the soluble protein fraction by *E. coli* grown at 25°C (Fig. S2C). The MtnB domain protein was purified by Ni^2+^-affinity chromatography, and the native molecular mass was estimated at approximately 160 kDa (molecular mass of a monomer = 26.3 kDa) by native-PAGE (Fig. S2D). This indicated that MtnB formed a hexamer, similar to the full-length MtnBD.

**Figure 4:**
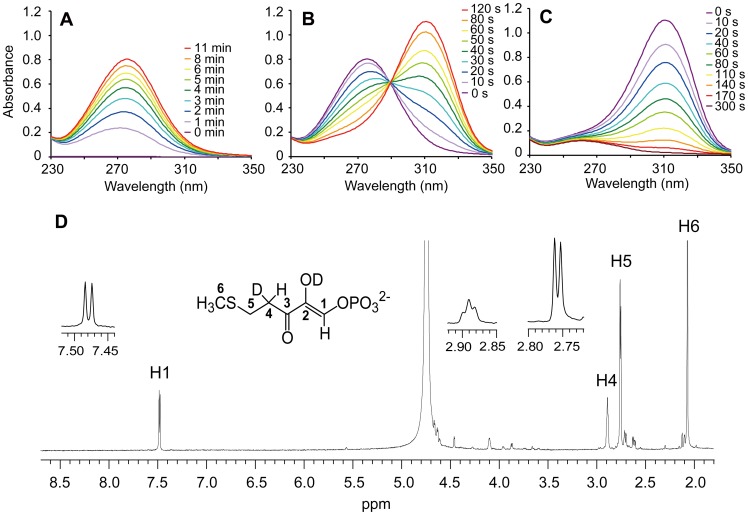
UV-visible and ^1^H-NMR spectra of products derived from the MtnB domain protein. (A) Spectral changes before and after addition of 2.4 µg MtnB domain protein to reaction mixture containing MTRu-1-P. Spectra are shown in different colors (0, 1, 2, 3, 4, 5, 6, 8, and 11 min). (B) Spectral shifts before and after adding 0.14 µg *B. subtilis* MtnX to reaction mixture in Fig. 4A. Spectra are shown in different colors (0, 10, 20, 30, 40, 50, 60, 80, and 120 s). (C) Spectral changes before and after adding 0.07 µg MtnBD to reaction mixture in Fig. 4B. Spectra are shown in different colors (0, 10, 20, 40, 60, 80, 110, 140, 170, and 300 s). All spectra were measured at pH 8.0 and 35°C. (D) ^1^H-NMR spectrum of products produced by 10 µg MtnB domain protein and MTRu-1-P. Reaction was performed for 2 h in 250 µL phosphate magnesium buffer (pD 7.5) at 25°C. The peak at 4.74 ppm shows H_2_O.

To analyze the catalytic reaction of the *T. thermophila* MtnB domain, we analyzed the UV-visible and ^1^H-NMR spectra of the reaction product from MTRu-1-P (Fig. 4). In the UV-visible spectrum, the reaction product showed maximum absorption at 278 nm, which is specific to HK-MTPenyl-1-P, the enolase product (Fig. 4A). In the ^1^H-NMR spectrum, a doublet peak at 7.48 ppm, a triplet peak at 2.89 ppm, and a doublet peak at 2.76 ppm corresponded to C1, C4, and C5 protons, respectively, the same spectrum as that of the MtnBD reaction product, HK-MTPenyl-1-P (Fig. 4D). These results demonstrated that the *T. thermophila* MtnB domain catalyzed the dehydration/enolization reactions to convert MTRu-1-P to HK-MTPenyl-1-P, the same as the reaction catalyzed by the full-length MtnBD. The MTRu-1-P dehydratase/enolase activity of the MtnB domain had a *k*_cat_ of 0.24±0.02 s^−1^ (mean ± S.E.), 26.4% of that of the full-length MtnBD (*k*_cat_ = 0.91±0.04 s^−1^) at pH 8.0 and 35°C. This lower *k*_cat_ might be because of the lack of the MtnD domain.

### The MtnD Domain Catalyzes the Dioxygenase Reaction of DHK-MTPene

**Figure 5:**
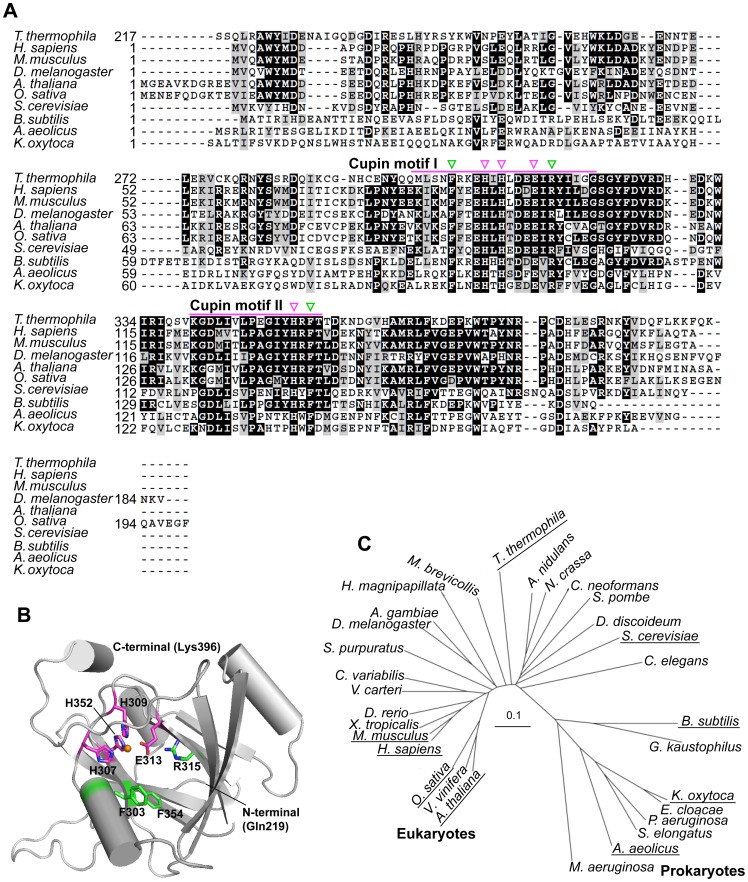
Sequence, structure, and phylogenetic analyses of various MtnDs. (A) Multiple sequence alignment of the MtnD family. Magenta bars show highly conserved cupin motifs I and II, magenta and green triangles show amino acid residues involved in metal binding and catalysis, respectively. (B) Predicted tertiary structure of *T. thermophila* MtnD. The structure of MtnD was modeled on that of *M. musculus* MtnD (PDB ID: 1VR3) using Swiss-model. Orange sphere represents a nickel atom. His307, His309, Glu313 and His352 (shown in magenta) form the metal binding site. Phe303, Arg315, and Phe 354 (shown in green) are essential for dioxygenation. (C) Phylogenetic tree based on primary sequences of the MtnD family. Alignments were created with ClustalW and displayed using Treeview. Scale bar indicates a difference of 0.1 substitutions per site. Names and gene accession numbers are as follows: *A. gambiae* (XP_315627), *A. aeolicus* (NP_214354), *A. thaliana* (NP_567443), *A. nidulans* (XP_664180), *B. subtilis* (NP_389245), *C. elegans* (NP_510072), *C. variabilis* (EFN58452), *C. neoformans* (XP_572521), *D. rerio* (NP_955962), *D. discoideum* (XP_635174), *D. melanogaster* (NP_001097577), *E. cloacae* 13047 (YP_003613573), *G. kaustophilus* (YP_146809), *H. sapiens* (NP_060739), *H. magnipapillata* (XP_002165167), *K. oxytoca* (AAD11793), *M. aeruginosa* (A8YMJ4), *M. brevicollis* MX1 (XP_001750499), *M. musculus* (NP_598813), *N. crassa* (XP_956660), *O. sativa* (AAC05511), *P. aeruginosa* (NP_250375), *S. cerevisiae* (NP_013722), *S. pombe* (NP_596475), *S. purpuratus* (XP_789320), *S. elongatus* (YP_171626), *V. vinifera* (CBI26309), *V. carteri* (XP_002955803), *X. tropicalis* (NP_001004933). Proteins described in (A) are underscored.

The sequence alignment of MtnDs from various organisms showed that the *T. thermophila* MtnD domain contained the conserved residues His307, His309, Glu313, and His352 to coordinate a metal ion (Fe^2+^ or Ni^2+^) essential for catalysis (Fig. 5A). Like other MtnDs reported previously [43]–[45], the *T. thermophila* MtnD domain also had conserved catalytic residues including Arg315, a candidate for deprotonation, and Phe303 and Phe354, which help to orient the substrate in the dioxygenase reaction. The MtnD domain had one predicted cupin domain, a small thermostable structure. It was composed of two β-strands (cupin motifs I and II) separated by a variable loop region, similar to other dioxygenases [46], [47] (Fig. 5A). Homology modeling of the MtnD domain using *M. musculus* MtnD (PDB ID: 1VR3) as the template suggested that these seven catalytic residues form an active site (Fig. 5B). In the phylogenetic tree, MtnDs were classified into prokaryotic and eukaryotic clades. The *T. thermophila* MtnD domain was in the eukaryotic clade (Fig. 5C). Sequence analysis together with prediction of MtnD structure strongly suggested that the MtnD domain catalyzes the DHK-MTPene dioxygenase reaction. Unfortunately, the MtnD domain was not expressed at all in the soluble fraction of *E. coli* grown at 25°C or 37°C (Fig. S2E). Therefore, the catalytic activity of the MtnD domain was evaluated using a combination of full-length MtnBD, the MtnB domain protein, and *B. subtilis* MtnX, by analyzing the UV-visible and ^1^H-NMR spectra of the reaction products. In the first step, DHK-MTPene was obtained from the reaction of *B. subtilis* MtnX with HK-MTPenyl-1-P produced by the MtnB domain (Fig. 4B). DHK-MTPene showed a specific UV-visible spectrum with maximum absorption at 310 nm. In the ^1^H-NMR spectrum, it showed a singlet peak at 8.5 ppm for C1 protons, a triplet peak at 2.73 ppm for C5 protons, and a doublet peak at 2.67 ppm for C4 protons (data not shown). This ^1^H-NMR spectrum showed that the MtnB domain and *B. subtilis* MtnX catalyzed the conversion from MTRu-1-P to DHK-MTPene, but not to KMTB. Thus, it was demonstrated that the MtnB domain did not catalyze the DHK-MTPene dioxygenase reaction. A decrease in absorption at 310 nm due to a decrease in DHK-MTPene occurred after adding full-length MtnBD to the reaction mixture containing DHK-MTPene produced by the MtnB domain and *B. subtilis* MtnX (Fig. 4C), similar to the DHK-MTPene dioxygenase reaction catalyzed by the *B. subtilis* MtnD [18]. These results revealed that the MtnD domain catalyzed the DHK-MTPene dioxygenase reaction.

**Figure 6:**
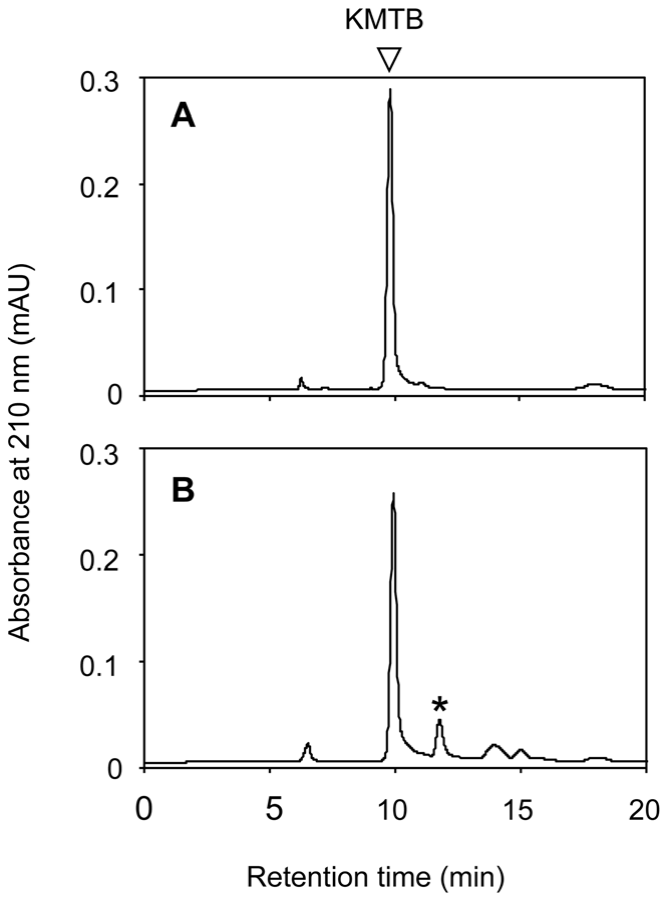
HPLC analyses of reaction products of dioxygenase reaction. (A) Authentic KMTB (1 mM). (B) Chromatogram of products generated by MtnBD and *B. subtilis* MtnX. The triangle represents KMTB. * indicates an unidentified peak at 11.8 min.

MtnDs from *B. subtilis*, *K. oxytoca*, and *Oryza sativa* are bi-functional enzymes that depend on metal coordination. The Fe^2+^-bound form catalyzes the production of KMTB as an on-pathway product, while the Ni^2+^-bound form of this enzyme catalyzes production of 3-hydroxypropionate as an off-pathway product (Fig. 1A) [18], [45]. The *T. thermophila* MtnBD and *B. subtilis* MtnX were incubated with MTRu-1-P as a starting substrate, and the reaction product was analyzed by HPLC after 15 h incubation (Fig. 6). The main peak (9.9 min retention time) was KMTB. Although it was unknown which metal ion bound to *T. thermophila* MtnBD in our preparation, this result clearly showed that MtnBD catalyzed the dioxygenase reaction to yield KMTB, as a precursor metabolite of methionine, from DHK-MTPene.

## Discussion

Our results highlighted that the MtnB domain of *T. thermophila* MtnBD catalyzes MTRu-1-P dehydratase/enolase reactions and the MtnD domain catalyzes the DHK-MTPene dioxygenase reaction. Notably, MtnB is a bi-functional domain with dehydratase and enolase activities. The MtnB domain is homologous to other MTRu-1-P dehydratases, and has three conserved histidine residues essential for the MTRu-1-P dehydration reaction (Fig. 3A, B). However, it does not have homologous domain for other enzymes (e.g., MtnW and MtnC) catalyzing the enolization of DK-MTP-1-P. In the conversion reaction from MTRu-1-P to HK-MTPenyl-1-P catalyzed by full-length MtnBD or the MtnB domain, DK-MTP-1-P was not observed in the ^1^H-NMR spectra (Fig. 2F, G, H and Fig. 4D). However, a deuterium was incorporated at the C4 position in D_2_O buffer during the reaction catalyzed by MtnBD or the MtnB domain (Fig. 2A, F, G, H and Fig. 4D). Incorporation of deuterium at the C4 position was also observed in the dehydratase reaction of *B. subtilis* MtnB [18], suggesting that the MtnB domain catalyzes sequential MTRu-1-P dehydratase and DK-MTP-1-P enolase reactions to produce HK-MTPenyl-1-P from MTRu-1-P. In the reaction catalyzed by the MtnB domain, it is likely that DK-MTP-1-P is not released from the active site after the MTRu-1-P dehydratase reaction and is immediately enolized in the active site cavity. This proposed mechanism is supported by the fact that DK-MTP-1-P was not detected in the reaction catalyzed by the MtnB domain in which MTRu-1-P is converted into HK-MTPenyl-1-P. It is reasonable to use one enzyme for the dehydratase and enolase reaction steps, because the reaction product of MTRu-1-P dehydratase, DK-MTP-1-P, is labile, and decomposes at a rate constant of 0.048 s^−1^, as described previously [30]. DK-MTP-1-P decomposition results from hydration of the 2-carbonyl group and hydrated DK-MTP-1-P may not be a substrate for the enolization reaction [48]. In *T. thermophila,* the occurrence of two sequential reactions in one active site in the MtnB domain may enhance their catalytic efficiencies, since this would avoid the spontaneous hydration of DK-MTP-1-P by water attack from the solvent if it was released from the active site during dehydratase and enolase reactions.

Warlick and co-workers reported that DK-MTP-1-P is enolized non-enzymatically to HK-MTPenyl-1-P with a rate constant of 0.0004 s^−1^ and *t*_1/2_ of ∼30 min for the initial formation of HK-MTPenyl-1-P [48]. In the case of *T. thermophila* MtnBD, 85.7 µM MTRu-1-P (with a calculated equilibrium constant for [MTRu-1-P]/[MTR-1-P] of 6.0) [20] was completely converted into HK-MTPenyl-1-P in 420 s (Fig. 2B). The rate of enolization catalyzed by MtnBD is much faster than that of non-enzymatic enolization. In addition, the formation rate of HK-MTPenyl-1-P depended on the amount of MtnBD protein (Fig. 2D). These results show that enolization is catalyzed enzymatically by MtnBD. However, the question of how the MtnB domain catalyzes enolization remains unanswered. The MtnB domain may have catalytic residues that deprotonate and reprotonate for enolization, like MtnW, the DK-MTP-1-P enolase [19], [31]. Further structural and mutational analyses are needed to reveal the catalytic mechanism of the enolase reaction catalyzed by the MtnB domain.

The MtnD domain of *T. thermophila* MtnBD may be required for efficient catalytic function of the MtnB domain, because deletion of the MtnD domain caused a 73.6% decrease in the MTRu-1-P dehydratase/enolase activity of the MtnB domain. The MtnD domain may be involved in forming an optimal active site in the MtnB domain. Both the MtnB domain and MtnBD are predicted to form homohexamers (Fig. S2D). Conversely, the MtnDs from *K. oxytoca* and *M. musculus* function as monomers [44], [49]. These facts suggest that MtnBD forms a hexamer with the MtnB domain. This conformation of the MtnB and MtnD domains may optimize the active site in each domain of the fusion enzyme.

Our results demonstrated that the *Tetrahymena* MtnBD does not catalyze the HK-MTPenyl-1-P phosphatase reaction (Fig. 2). This contradicts the previous suggestion that the *T. thermophila* MtnBD catalyzes MTRu-1-P dehydratase, DK-MTP-1-P enolase, HK-MTPenyl-1-P phosphatase, and DHK-MTPene dioxygenase reactions, as determined by complementation tests of yeast knockout mutants of *mtnB*, *mtnC,* and *mtnD* using the gene for *T. thermophila* MtnBD for rescue [14]. Why was the growth phenotype of the *mtnC* (encoding an enolase/phosphatase) yeast mutant complemented by the *T. thermophila mtnBD* gene? One possible explanation is that the yeast genome contains a gene for a MtnX (HK-MTPenyl-1-P phosphatase) sharing 28% amino acid sequence identity with the *B. subtilis* MtnX (Fig. S3). This MtnX homolog has three conserved catalytic motifs (I, II and III) for dephosphorylation, which is characteristic of the l-2-halo-acid dehalogenase hydrolase superfamily [37], [50] that includes MtnX and MtnC. The MtnX homolog may function as a HK-MTPenyl-1-P phosphatase in the MSP in yeast. Another explanation is the wide substrate specificity of phosphatases. For example, alkaline phosphatase can catalyze dephosphorylation of HK-MTPenyl-1-P (Nakano and Ashida, unpublished data). Various non-specific phosphatases may be involved in dephosphorylation in the MSP of yeast. A *B. subtilis* knockout mutant of *mtnX* grew slowly, but was able to survive in medium containing MTA as the sole source of sulfur [22]. This suggests that non-specific phosphatases may catalyze dephosphorylation of HK-MTPenyl-1-P *in vivo*, which supports our idea. Interestingly, *T. thermophila* also has a MtnX homolog (XP_001011136) showing 31% sequence identity with the *B. subtilis* MtnX (Fig. S3). Thus, phosphatases, including this MtnX homolog, probably catalyze the HK-MTPenyl-1-P phosphatase reaction *in vivo* in *T. thermophila.*

The MtnB domain has novel bi-functional enzyme activity, catalyzing both dehydratase and enolase reactions. It has been reported in other species that the DK-MTP-1-P enolase reaction is catalyzed by MtnW, which shows ∼25% sequence identity with ribulose-bisphosphate carboxylase/oxygenase [18], [19], [21], [40], [51], [52], or MtnC, a bi-functional enzyme catalyzing the DK-MTP-1-P enolase/phosphatase reaction [37], [50], [53]. Therefore, *T. thermophila* uses only the MtnB domain to catalyze dehydratase and enolase reactions, whereas other organisms use two separate enzymes, MtnB and MtnW or MtnB and MtnC, to catalyze these two reaction steps in the MSP. Thus, our study provides evidence for an economical MSP with only a few catalysts in *T. thermophila*. In the predicted MSP of *T. thermophila*, MTRu-1-P, the substrate of MtnBD, is synthesized from MTA by MtnN (XP_001012703) and MtnAK (XP_001031773). Subsequently, MTRu-1-P is converted to KMTB, the methionine precursor, by MtnBD and unknown phosphatase(s). KMTB is converted to methionine by KMTB aminotransferase, but this enzyme has not been identified yet in *T. thermophila.* The *T. thermophila* genome contains genes encoding homologs of the yeast aminotransferases Apo8, Apo9, Bat1, and Bat2 [14]. The *B. subtilis* knockout mutant of *mtnE* can grow in medium containing MTA as the sole sulfur source [22]. Therefore, it is predicted that the transamination of KMTB is catalyzed by KMTB aminotransferase candidates and non-specific aminotransferases in *T. thermophila.* Taken together, these results indicate that the *T. thermophila* MSP involves MtnN, MtnAK, MtnBD, and unidentified phosphatase(s) and aminotransferase(s).

To date, 34 genome projects have been completed for protozoa. However, MtnBD has been found in only two protozoa, namely *T. thermophila* and *Ichthyophthirius multifiliis*, which causes fish white-spot disease. This fusion protein is unique to these organisms, and is not found in other protozoa, including *Paramecium*, *Plasmodium*, *Trypanosoma*, *Leishmania,* and *Entamoeba*. The order of the domains is of particular interest; the MtnB domain is located at the N-terminus and the MtnD domain at the C-terminus. Interestingly, the order of the MSP genes is *mtnB* and *mtnD* in Bacilli. In fact, some Bacilli, including *B. subtilis*, *Geobacillus kaustophilus*, and *Anoxybacillus flavithermus*, have an *mtnWXBD* operon [23], [30]. In Gupta’s hypothesis of linear bacterial evolution, bacteria evolved from a common ancestor, and low G+C gram-positive bacteria such as Bacilli emerged first [54]–[56]. Based on Gupta’s proposal regarding bacterial evolution, the MSP of Bacilli (*mtnWXBD*) might be the most ancient and the origin of MSPs in other organisms. These hypotheses would suggest that the gene set and the order of *mtnB* and *mtnD* was transferred from an *mtnWXBD* operon of such bacteria to *T. thermophila* during evolution, and then the two genes combined to form a fusion protein. However, evolutionary selection of the original MSP in *T. thermophila* led to a distinct MtnBD that differs from enzymes in the MSPs of other organisms. This was likely caused by acquisition of MtnBD through the molecular evolution of the MtnB domain to gain an enolization function.

## Supporting Information

Figure S1 **Codon modification of the** ***mtnBD*** **gene from** ***Tetrahymena*** **.** Comparison of the *T. thermophila mtnBD* gene (XM_00102546) posted in the National Center of Biotechnology Information (NCBI) database with a codon modified *mtnBD* gene (syn_ttmtnBD). The multiple alignment was created using ClustalW 2.1 (http://www.genome.jp/tools/clustalw/).
(PDF)

Figure S2 **Constitution, expression, and purification of full-length MtnBD, MtnB domain, and MtnD domain proteins.** (A) Structures of full-length MtnBD, and MtnB and MtnD domain proteins for expression. Both the MtnB and MtnD domains have three histidine residues (shown by triangles) essential for catalysis. H at the N-terminus represents a histidine tag composed of 20 amino acids. SDS-PAGE analyses of soluble proteins from *E. coli* and purified MtnBD (B) and MtnB domain proteins (C); 8 µg soluble protein and purified enzyme were separated by denaturing gel electrophoresis. M: molecular markers. Calculated molecular masses of monomeric histidine-tagged MtnBD and MtnB domains are 48.4 and 26.3 kDa, respectively. (D) Native-PAGE analysis of MtnBD and MtnB domain proteins. Purified protein (1 µg) was separated on a 5–20% gradient gel under non-denaturing conditions. M: native molecular markers. (E) Expression of MtnD domain in *E. coli*. Histidine-tagged MtnD domain was overexpressed in *E. coli* BL21 (DE3) at 37°C and 25°C. Extracted total and soluble proteins (8 µg) from *E. coli* were analyzed by SDS-PAGE (12.5% polyacrylamide gels). Calculated molecular mass of the monomeric histidine-tagged MtnD domain is 24.4 kDa. All gels were stained with Coomassie brilliant blue R-250.
(PDF)

Figure S3 **Comparison of HK-MTPenyl-1-P phosphatase with its homologs.** Triangles show predicted active site residues. The alignment includes HK-MTPenyl-1-P phosphatases (MtnX) from *B. subtilis* (Genbank accession number NP_389243) and *G. kaustophilus* (YP_146807), HK-MTPenyl-1-P phosphatase homologs from *T. thermophila* (XP_001011136) and *S. cerevisiae* (NP_014388), phosphoserine phosphatase from *Methanococcus jannaschii* (NP_248603), and DK-MTP-1-P enolase/phosphatases (MtnC) from *O. sativa* (NP_001067908, C-terminal MtnC domain residues 253–518) and *H. sapiens* (NP_067027) containing motif I [DXDX(T/V)], motif II [(S/T)XX], and motif III [K-(X)n-(G/S)(D/S)XXX(D/N)] essential for catalysis in dephosphorylation and metal coordination. The multiple alignment was created manually and highlighted with Boxshade.
(PDF)
