# Supplementary material for: MtnBD Is a Multifunctional Fusion Enzyme in the Methionine Salvage Pathway of Tetrahymena thermophila
Source: PLoS One. 2013 Jul 1;8(7):e67385. doi: 10.1371/journal.pone.0067385 (PMC3698126; doi:10.1371/journal.pone.0067385)
Supplement: Figure S1 — Codon modification of the mtnBD gene from Tetrahymena . Comparison of the T. thermophila mtnBD gene (XM_00102546) posted in the National Center of Biotechnology Information (NCBI) database with a codon modified mtnBD gene (syn_ttmtnBD). The multiple alignment was created using ClustalW 2.1 (http://www.genome.jp/tools/clustalw/). (PDF) [file pone.0067385.s001.pdf]

Figure S1.

```
XM_001025046    1 ATGAACGGTTTATCTTCAAATTCTGGTCCTAGAGAAGTCATTTGTGCTCTCTTGAGAAAC
Syn_ttmtnBD     1 .....T...C.G..A..T..C........GC.T.....G...........GC..C.T..T

XM_001025046   61 TTTTATACTTAAGGATGGTGCGCTGGTTCAGGTGGTGGTATATCTATTAGAAAAAGCGAC
Syn_ttmtnBD    61 ........CC....C.....T.....C.....C.....C..TAGC..CC.T...TCT..T

XM_001025046  121 GATGAAATATATGTTGCACCTTCAGGTGTATAAAAAGAATTGGTTTAACCTGAAGATATC
Syn_ttmtnBD   121 ..C.....T........C..G........CC.G......C....GC....G.....C..T

XM_001025046  181 TATGTTATTAATGTTAATGGAGATGTAGTTGAAAACCCTAAAAATCCAAAATTAAAACCT
Syn_ttmtnBD   181 ..C..C..C..C..G.....C.....G...........G........G...C.G.....G

XM_001025046  241 TCTGAATGCACTCCTCTTTTTAATGCAGCTTATAAGCTTCGTCGTGCTGGTGCTGTTTTA
Syn_ttmtnBD   241 AGC........C..G..G.....C..G..C.....A..G.....C..A........CC.G

XM_001025046  301 CATTCTCATGCTCTTCCTGCAATGCTAGTTACCAAGCTCTTTGGAACTGAATTTTAGACA
Syn_ttmtnBD   301 ........C..G..G..G..C.....G..G..G..A..G.....T..C.....CC....G

XM_001025046  361 ATTGATCATGAAATGATTAAAGGTATTCCTAATCACCATAATACAGAATGGTGTGTCGTC
Syn_ttmtnBD   361 .......................C..C..G..C..T..C.....C........C.....G

XM_001025046  421 CCTATCATTGAAAACACTGAAAAGGAATGCGAATTAACAGAACGTTTAACTAATGCAATC
Syn_ttmtnBD   421 ..G..T..C........G.....A.....T...C.G..C.....CC.G..G..C......

XM_001025046  481 AACGCTTATCCTCGTTCTAATGCTGTCTTAGTTCGTAATCACGGTGTTTATATTTGGGGA
Syn_ttmtnBD   481 ..T........G..C..C..C..G..GC.G...........T........C........C

XM_001025046  541 GAAAATTGGGAAAAAGCAAAAATACATGCTGAGTGTTACCATTATTTATTCGAAGCAGTT
Syn_ttmtnBD   541 .................G.....C.....C..A..C..T..C..CC.G........C...

XM_001025046  601 GTTGAAATGAAGAAATTAGGTTTAGAAATTCCACGCACTGTTTCAAGCAGTAGCCAATTA
Syn_ttmtnBD   601 ..C........A...C.G..CC.G........G.....C..GAGCTCT...TC...GC.G

XM_001025046  661 CGTGCTTGGTATATTGATGAAAATGCAATTGGCCAAGATGGTGATATTCGTGAGAGCTTA
Syn_ttmtnBD   661 .....A.................C..T..C..T........C..C.....C..A..TC.G

XM_001025046  721 CACTATCGCTCATATAAGTGGGTTAATCCAGAATATTTGGCAACAATTGGTGTTGAACAC
Syn_ttmtnBD   721 ..T.....T..C..C..A.....G.....G......C.......C..C............

XM_001025046  781 TGGAAATTAGACGGAGAAGAGAATAATACTGAACTTGAAAGAGTTTGCAAATAAAGAAAT
Syn_ttmtnBD   781 ......C.G.....C.....A..C.....G.....G...C.T.....T...C.GC.C..C

XM_001025046  841 TACTCTTCTCGTGATTAAATTAAATGTGGAAATCATTGTGAAAATTACTAACAAATGTTA
Syn_ttmtnBD   841 ..T..A..G......C....C.....C..T..C...........C...C.G......C.G

XM_001025046  901 TCAAATTTCAGAAAAGAACACATCCATTTAGATGAAGAGATTCGCTATATTATAGGTGGT
Syn_ttmtnBD   901 AGC.....TC.C........T..T..CC.G........A..C..T........C.....C

XM_001025046  961 AGTGGTTATTTTGATGTCCGTGACCATGAAGATAAATGGATTCGTATCCAAAGTGTTAAA
Syn_ttmtnBD   961 TCA..C..C..C.....T..C.....C.......................GTCG......

XM_001025046 1021 GGTGATTTAATTGTACTTCCTGAAGGTATATATCACCGCTTTACAACTGATAAAAATGAT
Syn_ttmtnBD  1021 .....CC.G.....C..G..G.....C..C.....T........C..G..C.....C...

XM_001025046 1081 GGAGTTCATGCTATGAGATTATTTAAGGATGAACCTAAATGGACACCTTATAATCGTCCT
Syn_ttmtnBD  1081 ..T..G..C..G...C.TC.G..C..A........G........C..G..C.....C..G

XM_001025046 1141 TGTGATGAATTGGAAAGCCGTAACAAATATGTAGATTAATTCTTAAAAAAGTTTTAAAAA
Syn_ttmtnBD  1141 .....C...C.............T........T..CC.....C.G.....A..CC.....

XM_001025046 1201 TGA
Syn_ttmtnBD  1201 .A.
```
