# Supplementary figures and images for: MtnBD Is a Multifunctional Fusion Enzyme in the Methionine Salvage Pathway of Tetrahymena thermophila
Source: PLoS One. 2013 Jul 1;8(7):e67385. doi: 10.1371/journal.pone.0067385 (PMC3698126; doi:10.1371/journal.pone.0067385)

Figure S2.

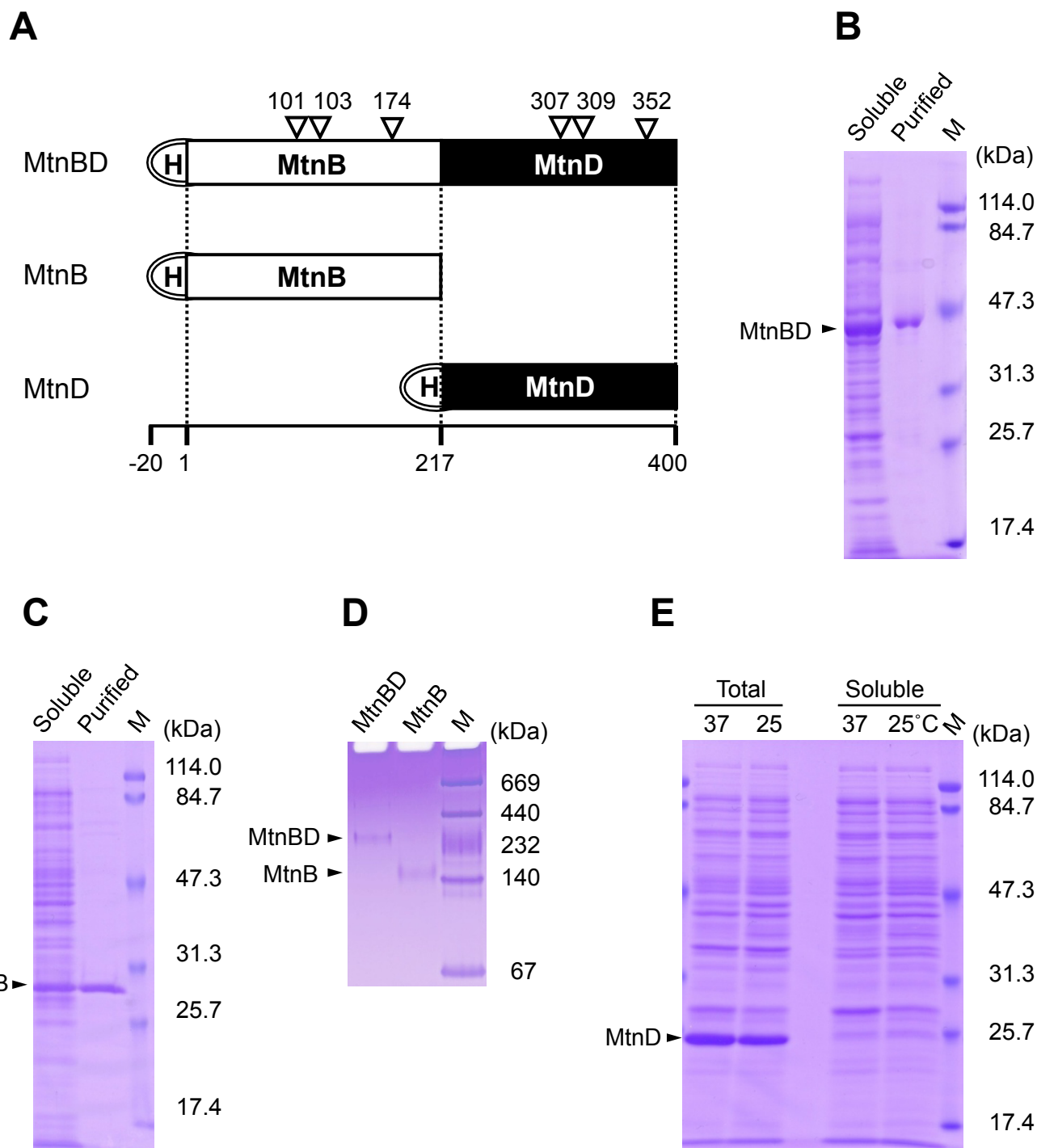

Supplement: Figure S2 — Constitution, expression, and purification of full-length MtnBD, MtnB domain, and MtnD domain proteins. (A) Structures of full-length MtnBD, and MtnB and MtnD domain proteins for expression. Both the MtnB and MtnD domains have three histidine residues (shown by triangles) essential for catalysis. H at the N-terminus represents a histidine tag composed of 20 amino acids. SDS-PAGE analyses of soluble proteins from E. coli and purified MtnBD (B) and MtnB domain proteins (C); 8 µg soluble protein and purified enzyme were separated by denaturing gel electrophoresis. M: molecular markers. Calculated molecular masses of monomeric histidine-tagged MtnBD and MtnB domains are 48.4 and 26.3 kDa, respectively. (D) Native-PAGE analysis of MtnBD and MtnB domain proteins. Purified protein (1 µg) was separated on a 5–20% gradient gel under non-denaturing conditions. M: native molecular markers. (E) Expression of MtnD domain in E. coli. Histidine-tagged MtnD domain was overexpressed in E. coli BL21 (DE3) at 37°C and 25°C. Extracted total and soluble proteins (8 µg) from E. coli were analyzed by SDS-PAGE (12.5% polyacrylamide gels). Calculated molecular mass of the monomeric histidine-tagged MtnD domain is 24.4 kDa. All gels were stained with Coomassie brilliant blue R-250. (PDF) [file pone.0067385.s002.pdf]

Figure S3.

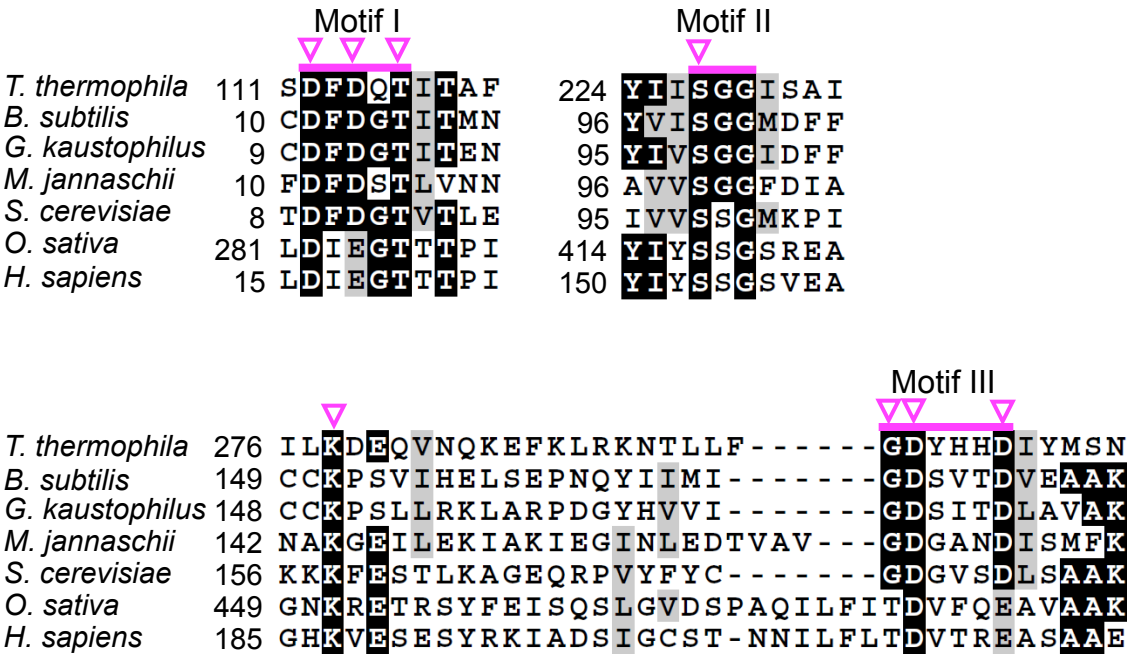

Supplement: Figure S3 — Comparison of HK-MTPenyl-1-P phosphatase with its homologs. Triangles show predicted active site residues. The alignment includes HK-MTPenyl-1-P phosphatases (MtnX) from B. subtilis (Genbank accession number NP_389243) and G. kaustophilus (YP_146807), HK-MTPenyl-1-P phosphatase homologs from T. thermophila (XP_001011136) and S. cerevisiae (NP_014388), phosphoserine phosphatase from Methanococcus jannaschii (NP_248603), and DK-MTP-1-P enolase/phosphatases (MtnC) from O. sativa (NP_001067908, C-terminal MtnC domain residues 253–518) and H. sapiens (NP_067027) containing motif I [DXDX(T/V)], motif II [(S/T)XX], and motif III [K-(X)n-(G/S)(D/S)XXX(D/N)] essential for catalysis in dephosphorylation and metal coordination. The multiple alignment was created manually and highlighted with Boxshade. (PDF) [file pone.0067385.s003.pdf]
